# Oxidative Stress and the ER Stress Response in a Murine Model for Early-Stage Alcoholic Liver Disease

**DOI:** 10.1155/2012/207594

**Published:** 2012-07-05

**Authors:** James J. Galligan, Rebecca L. Smathers, Colin T. Shearn, Kristofer S. Fritz, Donald S. Backos, Hua Jiang, Christopher C. Franklin, David J. Orlicky, Kenneth N. MacLean, Dennis R. Petersen

**Affiliations:** ^1^Department of Pharmacology, University of Colorado Anschutz Medical Campus, Aurora, CO 80045, USA; ^2^Department of Pharmaceutical Sciences, University of Colorado Anschutz Medical Campus, Aurora, CO 80045, USA; ^3^Department of Pediatrics, University of Colorado Anschutz Medical Campus, Aurora, CO 80045, USA; ^4^Department of Pathology, University of Colorado Anschutz Medical Campus, Aurora, CO 80045, USA

## Abstract

Alcoholic liver disease (ALD) is a primary cause of morbidity and mortality in the United States and constitutes a significant socioeconomic burden. Previous work has implicated oxidative stress and endoplasmic reticulum (ER) stress in the etiology of ALD; however, the complex and interrelated nature of these cellular responses presently confounds our understanding of ethanol-induced hepatopathy. In this paper, we assessed the pathological contribution of oxidative stress and ER stress in a time-course mouse model of early-stage ALD. Ethanol-treated mice exhibited significant hepatic panlobular steatosis and elevated plasma ALT values compared to isocaloric controls. Oxidative stress was observed in the ethanol-treated animals through a significant increase in hepatic TBARS and immunohistochemical staining of 4-HNE-modified proteins. Hepatic glutathione (GSH) levels were significantly decreased as a consequence of decreased CBS activity, increased GSH utilization, and increased protein glutathionylation. At the same time, immunoblot analysis of the PERK, IRE1**α**, ATF6, and SREBP pathways reveals no significant role for these UPR pathways in the etiology of hepatic steatosis associated with early-stage ALD. Collectively, our results indicate a primary pathogenic role for oxidative stress in the early initiating stages of ALD that precedes the involvement of the ER stress response.

## 1. Introduction

As the predominant cause of liver injury, alcoholic liver disease (ALD) remains a major cause of both morbidity and mortality in the Western world. Alcohol consumption in developing countries is increasing significantly and ALD is becoming a major socioeconomic burden. In 2003, it was estimated that alcohol was responsible for 44% of all deaths from liver disease [1]. Despite its overwhelming prevalence, only 10–15% of alcoholics ever develop hepatitis and cirrhosis [2]. As a result, it is crucial to intervene therapeutically during the early initiating stages of the disease where recovery is possible. ALD is a multifactorial disease, resulting from numerous cellular derangements, creating difficulties when trying to develop a targeted therapy [3]. One of the major confounding factors in our understanding of the pathogenesis of ALD is the generation of reactive oxygen species (ROS) resulting from the oxidative metabolism of ethanol. 

A well-documented consequence of ethanol ingestion is the induction of cytochrome P450s, specifically CYP2E1, and depletion of cellular antioxidants leading to a prooxidative state [4]. While enhanced generation of ROS is likely to occur through multiple mechanisms, CYP2E1 and NADPH oxidase (Nox) are thought to be major sources [2, 4, 5]. Sustained exposure to ROS leads to prolonged oxidative stress, which promotes lipid peroxidation and generation of reactive aldehydes, such as 4-hydroxy-2-nonenal (4-HNE). This highly reactive aldehyde has been shown to covalently modify numerous cellular proteins, often resulting in alterations in protein structure, localization, function, and/or activity [6]. 

Protein folding is a critical function of the hepatic ER, yet despite the presence of a large number of chaperone proteins in the ER, an estimated 30% of nascent proteins fail to reach their properly folded state [7, 8]. Given this high frequency of improper folding, the ER incorporates a quality control system known as ER-associated degradation (ERAD), which eliminates unwanted proteins through ubiquitination and subsequent proteasomal degradation [9]. Under situations of sustained stress, where ERAD processes cannot eliminate all unfolded/misfolded proteins, the cell undergoes a series of adaptive signaling events termed the unfolded protein response (UPR). The overall goal of UPR signaling is to selectively increase the transcription of chaperones and increase ERAD processes, while decreasing overall translation [10]. UPR signaling is also known to induce selective signaling associated with both lipid and cholesterol homeostasis through activation of the sterol regulatory element-binding proteins (SREBPs) [11, 12]. These transcription factors tightly regulate fatty acid synthesis (SREBP-1) and cholesterol synthesis (SREBP-2). Previous studies by Ji et al. have implicated a role for the UPR in a rodent model of ALD [13, 14]. Although the proposed mechanism of this ALD-associated UPR induction is through increased plasma homocysteine, others have suggested a prominent role for oxidative stress in the activation of the UPR [8, 15–17]. In addition, sustained UPR activation has been shown to lead to enhanced ROS generation resulting in oxidative stress through activation of the ER oxidoreductases (Ero1) and NAD(P)H oxidase 4 (Nox4) [17, 18]. 

Altered redox control in the ER is linked to numerous hepatic disease states, including ALD [15, 19]. The transition from steatosis to steatohepatitis is considered to be the critical pathogenic determinant in ALD, furthering a need for therapeutic intervention during the early initiating steatotic stages of disease progression [20]. The mechanisms behind this transition remain elusive, and research on ALD is rarely focused on this stage. In this paper, we demonstrate a clear role for oxidative stress in the initiation of ALD that precedes the involvement of the UPR. These findings show, for the first time, a clear dissociation between oxidative stress and UPR signaling in the initiation of ALD. 

## 2. Experimental Procedures

### 2.1. Animal Model

 All procedures involving animals were approved by the Institutional Animal Care and Use Committee of the University of Colorado and were performed in accordance with published National Institutes of Health guidelines. Male C57/BL6J mice (12 per group) were utilized for the analysis and characterization of ethanol-mediated liver damage. Briefly, mice were fed a modified Lieber-DeCarli liquid-based diet (Bio-Serv, Frenchtown, NJ) for a period of 6 wk. The diet consisted of 44% fat-derived calories and 16% protein-derived calories, and the remaining balance was comprised of either maltose-dextrin or ethanol-derived calories. Ethanol-fed mice began the study on a diet consisting of 2% (v/v) ethanol, with the ethanol-derived calories increasing on a weekly basis until sacrifice; week 6 consisted of 6% ethanol (v/v) or 31.8% ethanol-derived calories. Pair-fed control animals received the remaining caloric intake from carbohydrate source (the caloric content of the diet used for the ethanol administration paradigm is presented in Table 1). Food consumption was monitored daily, and body weights were measured once per week. Upon completion of the study, animals were anesthetized via intraperitoneal injection with sodium pentobarbital and euthanized by exsanguination. Blood was collected from the inferior vena cava, and plasma was separated through centrifugation and assayed for alanine aminotransferase (ALT) activity (Diagnostic Chemicals Limited, Oxford, CT). Livers were excised, weighed, and frozen for biochemical characterization or subjected to differential centrifugation for subcellular fractionation as previously described [21].

As a positive control, endoplasmic reticulum stress was induced in C57BL/6J mice (7–11 wks) by a single IP injection with tunicamycin (Tm) at 0.5 mg/kg body weight [22]. Liver tissue was harvested 72 hours after injection.

### 2.2. Biochemical Analysis

Liver triglycerides were measured using a 2 : 1 chloroform : methanol extraction from liver homogenates using a kit from Diagnostic Chemicals Limited. Protein concentrations were determined using a BCA Protein Assay from Pierce (Rockford, IL) or a modified Lowry Protein Assay from Bio-Rad (Hercules, CA). Thiobarbituric acid reactive substances (TBARSs) were measured in whole liver homogenates (500 *μ*g) by incubating samples in thiobarbituric acid reagent (15% w/v trichloroacetic acid, 0.375% w/v thiobarbituric acid, and 0.25 N HCl). Samples were boiled at 100°C for 15 min, cooled to room temperature, and then centrifuged at 10,000 rpm. Absorbance of the cleared supernatant was read spectrophotometrically on a microplate reader at 535 nm, and the concentration of malondialdehyde (MDA) was calculated from the extinction coefficient, *ε* = 156,000 M^−1^ cm^−1^. The activity of glutathione reductase (GR) was determined in cytosolic fractions according to Babu et al. [23] Glutathione S-transferase (GST) activity was measured in whole liver homogenates according to Rinaldi et al. [24]. Glutathione peroxidase (GPx) activity was determined in whole liver homogenates using a commercially available kit (Cayman Chemical, Ann Arbor, MI). The enzymatic activity of glutaredoxin (GRx) was determined using the HEDS assay according to Mesecke et al. [25].

### 2.3. Quantification of Blood Ethanol Concentration (BEC)

BEC was determined as previously described [26]. Briefly, blood was collected on a weekly basis from the submandibular area of the mouse and placed in a heparinized tube; 0.6 M perchloric acid was added to each tube to precipitate protein. Samples were then centrifuged at 20,000 ×g for 10 minutes, and isopropanol was added to each tube as the internal standard. Samples were capped with air-tight rubber septums and heated for 15 min at 60°C. Head space gas was withdrawn (0.2 mL) and injected with a gas-tight syringe into a Hewlett-Packard 5710A gas chromatograph (injection port: 150°C, detector: 250°C, oven: 85°C). Separation of ethanol and isopropanol was performed on a 6 ft column packed with 60/80 Carbopack B (Supelco, Inc., Bellefonte, PA). Nitrogen was used as the carrier gas (flow rate = 40 mL/min). Ethanol concentrations were quantified against a standard curve using isopropanol as an internal standard to control for injection volume variation.

### 2.4. Cystathionine *β*-Synthase (CBS), Cystathionine *γ*-Lyase (CGL), and Glutamate-Cysteine Ligase (GCL) Measurements

Mouse livers were homogenized in buffer containing 100 mM potassium phosphate, pH 7.4, 1 mM EDTA, and 1 : 50 (v/v) protease inhibitor cocktail (Sigma Aldrich). The ratio of liver tissue to lysis buffer was 1 : 5. The homogenate was subsequently centrifuged at 4°C at 20,000 ×g for 20 min. The resultant supernatant was used as a crude extract. Protein concentration of the crude extract was determined by the Bradford method using bovine serum albumin as a standard. CBS activity assays were carried out using modifications to the method of Kashiwamata and Greenberg with a ninhydrin reagent specifically targeting cystathionine as described by Villanueva et al. [27, 28]. Briefly, 700 *μ*g of crude liver extract protein was incubated in assay buffer containing 100 mM Tris-HCl, pH 8.6, 10 mM L-serine, 1 mM pyridoxal 5-phosphate, and 2 mM DL-propargylglycine at 37°C for 5 min. L-homocysteine was then added to a final concentration of 26 mM and a final volume of 50 *μ*L to start the reaction at 37°C for 1 h. The reaction was stopped by the addition of 50 *μ*L of 10% trichloroacetic acid (TCA). Samples were subsequently centrifuged at 20,000 ×g for 5 min, and a 50 *μ*L sample of the supernatant was taken for cystathionine quantification as described previously [27]. Duplicate reactions were run in the absence of 10 mM L-serine as a negative control for each sample. CGL activity assays were performed using an enzyme-coupled assay with lactic dehydrogenase (LDH) as described previously [29]. Assay reactions were preincubated at 37°C and contained 100 mM potassium phosphate, pH 8.0, 2 mM L-cystathionine, 175 mM NADH, and five units of LDH. Reactions were started by the addition of 25 *μ*g of crude liver extract to give a final reaction volume of 100 *μ*L. The oxidation rate of NADH was monitored at 340 nm for 2 min at 37°C as an index of CGL activity. GCL activity was assayed from 1.0 mg of whole liver extract utilizing previously described protocols [30].

### 2.5. Measurement of Reduced and Oxidized Glutathione

Glutathione measurements were performed using a modified method from Reed et al. [31]. Briefly, fresh liver tissue was homogenized in 0.1 N perchloric acid using a sonicator and centrifuged at 13,000 rpm for 15 min, and the cleared supernatant was further neutralized with sodium bicarbonate and treated with 1 : 10 iodoacetic acid (IAA) (14.9 mg/mL) in the dark for 45 min at room temperature. Samples were then derivatized (1 : 1) with Sanger's reagent (1.5% 1-fluoro-2,4-dinitrobenzene (DFNB) in absolute ethanol) overnight in the dark at room temperature. Derivatized samples were stored at 4°C in the dark until analyzed by HPLC. Samples were applied (50 *μ*L injection) to a Supelco LC-NH_2_ 5 *μ*m, 25 cm × 4.1 mm column (Sigma Aldrich, St. Louis, MO) separated with a gradient mobile phase system. Solvent A (80% methanol) and Solvent B (80% Solvent B, 20% acetic acid/ammonium acetate solution (756 mL glacial acetic acid, 244 mL water, and 308 g of ammonium acetate)) were used for the separation of derivatized reduced (GSH) and oxidized (GSSG) glutathione using a Shimadzu LC-10AD dual pump system (flow rate of 1.0 mL/min) coupled to a Shimadzu SPD-M10AV diode array detector (Kyoto, Japan) set to 350 nm. HPLC running conditions were as follows: isocratic period of 50% solvent B for 10 min, followed by a linear gradient from 50% solvent B to 95% solvent B for 15 min, for a total run time of 35 min. Typical elution times were 11.55 min for GSH and 17.05 min for GSSG. Nanomolar concentrations of GSH and GSSG were calculated against a standard curve of 7 points for each molecule.

### 2.6. Western Blotting

Proteins from either whole liver extracts or subcellular fractions were subjected to standard SDS-PAGE or native PAGE. Native PAGE was employed to analyze the relative levels of monomeric GCLC and GCL holoenzyme. Native samples were mixed with loading buffer lacking 2-mercaptoethanol and were not boiled prior to loading on 8% polyacrylamide gels without a stacking gel or SDS. All gels were transferred to a Hybond-P membrane (GE Healthcare, Buckinghamshire, UK) and then blocked for 30 min with a tris-buffered saline solution containing 1% Tween-20 (TBST) and 5% nonfat dry milk (NFDM). Membranes were probed with primary antibodies directed against inositol-requiring enzyme 1 (IRE-1) and its phosphorylated form (p-IRE1), eukaryotic translation initiating factor 2*α* (eIF2*α*), growth-arrest-and-DNA-damage-inducible gene 153 (GADD153, also known as CHOP), SREBP1, SREBP2, Nox-4, Lamin B1, activating transcription factor 6 (ATF6) (Abcam, Cambridge, MA), KDEL (GRP78 and GRP94) (Stressgen, Ann Arbor, MI), Ero1*α*  and Ero1*β* (ProteinTech Group, Chicago, IL), p-eIF2*α*  (Epitomics, Burlingame, CA), *β*-actin (Sigma), and GCLC and GCLM (antibodies were generously provided by Dr. Terrance Kavanagh, University of Washington, Seattle, WA). A horseradish peroxidase conjugated secondary (Jackson Labs, Bar Harbor, ME) was then applied and membranes were developed using ECL-Plus Reagent from GE Healthcare. Chemiluminescence was visualized using a Storm 860 scanner from Molecular Dynamics (Sunnyvale, CA).

### 2.7. Immunohistochemistry

Following excision, livers were sectioned and placed in 10% neutral buffered formalin for 8 h. Samples were processed, imbedded in paraffin, and mounted on slides by Colorado HistoPrep (Fort Collins, CO). One pair of slides was stained with hematoxylin and eosin (H&E) for histological characterization while the remaining slides were subject to deparaffinization and rehydration for immunohistochemical characterization using either custom antibodies generated in our laboratory directed against 4-HNE modified proteins, anti-CYP2E1 (Calbiochem, San Diego, CA), or anti-protein-SSG (ViroGen, Watertown, MA). 

### 2.8. Measurement of Proteasome Activity

Liver samples from control and ethanol-treated mice were homogenized in 0.1 M Tris-HCl and 0.25 M sucrose. Homogenates were centrifuged at 105,000 ×g for 1 h, and the resulting supernatant (cytosolic fraction) was assayed for chymotrypsin-like activity utilizing a commercially available kit (BioVision, San Francisco, CA). Values were calculated against a standard curve and are reported as a % of control-fed mice.

### 2.9. Image Analysis

Images of H&E or immunohistochemically stained liver sections were captured on an Olympus BX51 microscope equipped with a four-megapixel Macrofire digital camera (Optronics, Goleta, CA) using the PictureFrame Application 2.3 (Optronics, Goleta, CA). All images were processed by Photoshop (Adobe Systems Inc. Mountain View, CA).

### 2.10. Statistical Analysis

Statistical analysis and generation of graphs were performed using GraphPad Prism 4.02 (GraphPad Software, San Diego, CA). Differences between control and ethanol-fed mice were assessed using a paired Student's *t*-test. Differences were considered significant if **P* < 0.05*.

## 3. Results

### 3.1. Biochemical Characterization of Early-Stage ALD

As shown in Table 1, compared to their isocaloric controls, ethanol-fed mice displayed a decrease in overall body weight at each time point with the ethanol-treated mice weighing 16% less (*P* < 0.001) at the conclusion of the study (6 weeks). As outlined, the ethanol concentration was increased incrementally throughout the course of the study. Blood ethanol concentration (BEC) was monitored and displayed a consistent increase at each time point, with maximum concentrations observed at week 6 (245.383 mg/dL ± 33.747). Consistent with early-stage ALD, a significant increase in liver/body weight ratio was observed (Table 2). This phenomenon was observed as early as 3 weeks, with the largest difference observed following 6 weeks. To assess liver damage, plasma ALT values were determined at each time point; ethanol-fed mice displayed greater than a two-fold increase in plasma ALT levels by week 6, with no significant difference being observed at weeks 1 and 3. Given these parameters, it was determined that mice treated for 6 weeks with ethanol displayed the earliest significant signs of ethanol-induced liver injury, demonstrating pathologies consistent with early-stage ALD. 

### 3.2. Continuous Ethanol Ingestion Results in Steatosis and Lipid Peroxidation

**Figure 1:**
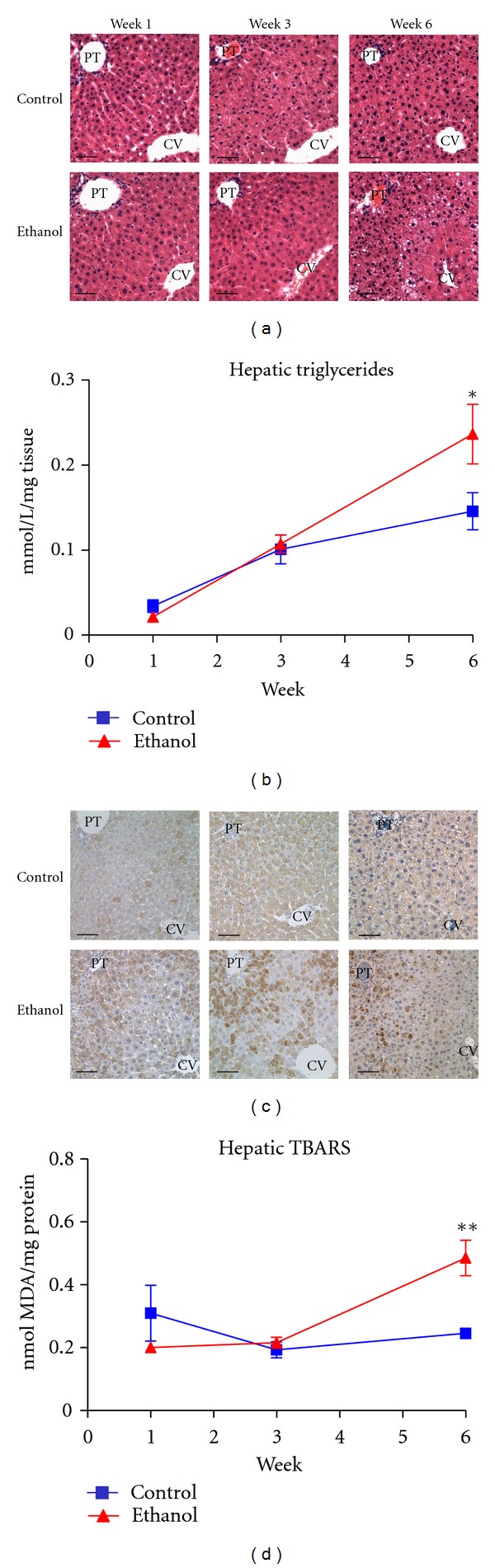
Chronic ethanol ingestion leads to increased hepatic lipid accumulation and lipid peroxidation. (a) H&E staining reveals marked pan-lobular lipid accumulation following 6 weeks of ethanol consumption. (b) Liver triglycerides are significantly increased following 6 weeks of ethanol ingestion. (c) Immunohistochemical analysis with 4-HNE antibodies reveals increased staining in ethanol-fed mice throughout zones 1 and 2 as early as week 3. (d) Hepatic TBARSs are significantly elevated following 6 weeks of ethanol ingestion. Magnification: 400x. Scale bar represents approximately 50 *μ*m. PT: portal triad; CV: central vein (**n* = 12*, *P < 0.05, **P < 0.01).

Hepatic steatosis is among the most prevalent and predictable outcomes of chronic ethanol consumption [3]. As shown in Figure 1(a), an increase in lipid was observed histologically as early as week 3 (here seen as very small lipid vesicles in zone 2), with a marked accumulation in lipid seen at week 6 (here involving both zones 1 and 2). Triglyceride content was also quantified at each time point; as shown in Figure 1(b), only the week 6 ethanol-fed-mice displayed a significant increase in hepatic triglycerides.

The increased accumulation of lipid observed in ALD provides the ideal environment for the generation of lipid aldehydes resulting from oxidative stress [32, 33]. As shown in Figure 1(c), immunohistochemical analysis reveals a substantial increase in hepatic staining of 4-HNE-modified proteins in as little as 3 weeks of feeding. The largest difference in staining, however, appeared following 6 weeks of ethanol ingestion with heavily stained hepatocytes localized predominately to zones 1 and 2. To validate this observation, hepatic TBARS was also quantified and is presented in Figure 1(d). Agreeing with the immunohistochemical staining in Figure 1(c), a significant increase in TBARS was observed at the week 6 time point, displaying nearly a two-fold increase. Collectively, these parameters indicate pathologies consistent with early-stage ALD, demonstrating a significant increase in hepatic lipid content and oxidative stress indices.

### 3.3. Effects of Ethanol Ingestion on GSH Synthesis and Utilization

As noted, decreased antioxidant capacity has been proposed to play a major role in the unbalanced generation of reactive aldehydes during ALD [6]. In line with this proposed mechanism, we sought to investigate the effects of ethanol consumption on hepatic GSH content and biosynthesis. As shown in Table 3, a significant depletion in reduced GSH was observed in mice treated for 6 weeks with ethanol compared to their respective isocaloric controls, indicating a decrease in the total antioxidant capacity of the liver at this time point. Oxidized GSH (GSSG) was also measured, observing no significant alterations at all time points analyzed. The reduction of GSSG to GSH is catalyzed by glutathione reductase (GR) and is thought to be the predominant mechanism for GSH cycling [34]. As shown in Table 3, GR activity was found to be significantly increased at the week 6 time point, with no variation observed at week 1 or week 3. This phenomenon has been observed in other models for oxidative stress and has been proposed to be a compensatory mechanism [28].

We and others have shown that oxidative stress and products of lipid peroxidation can also alter GSH biosynthesis via actions on GCL. This has been shown to occur through posttranslational modification of preexisting protein and/or changes in the relative levels of GCL holoenzyme, which has a significantly greater specific activity than monomeric GCLC [30, 35]. As shown in Table 3, the activity of GCL was significantly increased following 1 and 6 weeks of ethanol consumption. Standard immunoblotting revealed that ethanol ingestion had no significant effect on GCLC or GCLM expression (data not shown). Analysis of GCL holoenzyme formation by native gel electrophoresis and immunoblotting revealed no significant effect of ethanol ingestion on the relative levels of GCL holoenzyme compared to monomeric GCLC (data not shown). While these findings suggest that ethanol-induced oxidative stress increases hepatic GCL activity, it should be noted that these GCL activity measurements were performed under saturating substrate concentrations and thus reflect optimal GCL enzymatic activity. Importantly, such measurements do not take into account the potential contribution of altered substrate availability.

 Indeed, cysteine availability is often the rate-limiting factor in GSH biosynthesis. To assess endogenous cysteine synthesis and its effects on GSH biosynthesis, the transsulfuration pathway was investigated, specifically cystathionine beta synthase (CBS). As shown in Table 3, a significant decrease in CBS activity was observed at the week 6 time point (*P* < 0.001). This is likely a result of a significant decrease in protein expression (~30%, *P* < 0.05, data not shown) as a result of ethanol ingestion. The activity and expression of the transsulfuration enzyme cystathionine *γ*-lyase was also assessed and displayed no significant alterations in either parameter. Collectively, these data suggest that not only alcohol consumption leads to the increased utilization of GSH as an antioxidant but also the endogenous synthesis of the GSH precursor cysteine is significantly altered, providing a dual mechanism for the observed decrease in GSH.

### 3.4. Ethanol Ingestion Leads to Increased Protein Glutathionylation

**Figure 2:**
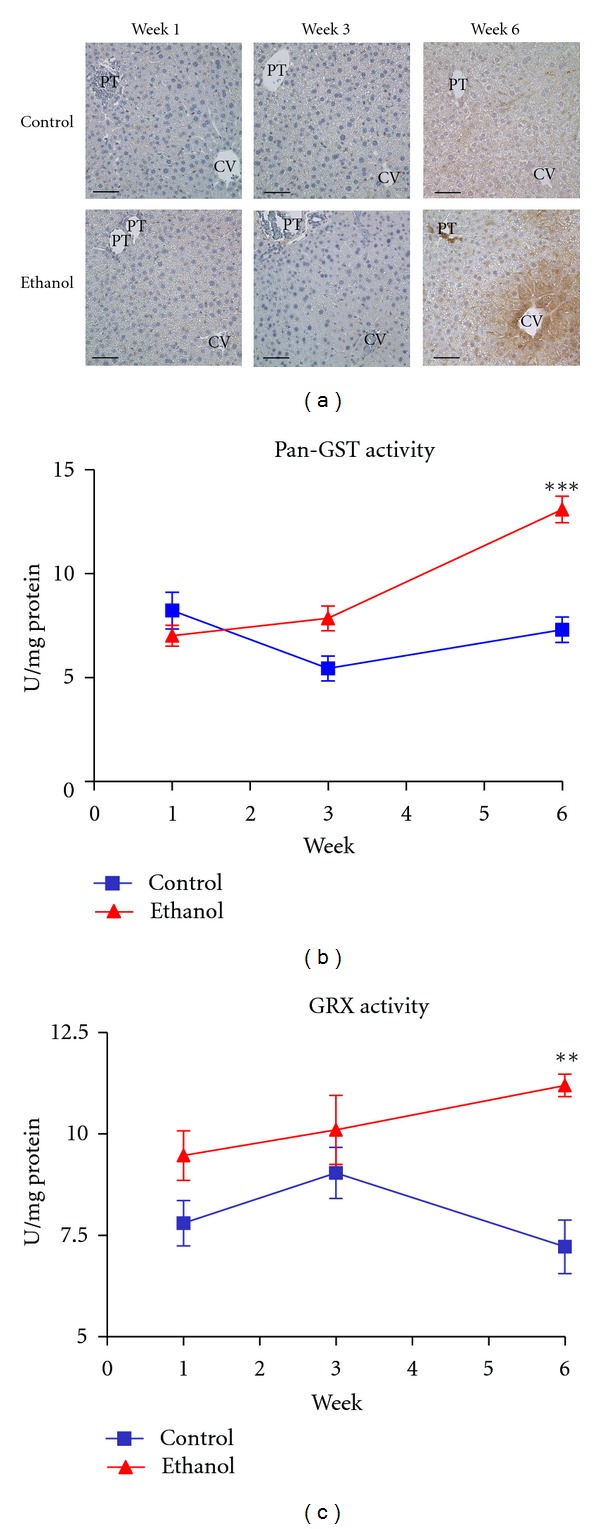
Ethanol ingestion results in increased protein-SSG. (a) Immunohistochemical staining with antibodies directed against protein-SSG reveals a marked increase in the zone 3 staining in the week 6 ethanol-fed mice. (b) pan-GST activity and (c) GRx activity significantly elevated at week 6. Magnification: 400x. Scale bar represents approximately 50 *μ*m. PT: portal triad: CV: central vein (*n* = 6, **P < 0.01, ***P < 0.001).

The binding of GSSG to protein thiols is referred to as protein glutathionylation (protein-SSG) and has been linked to numerous disease states associated with oxidative stress [36]. We, therefore, sought to investigate this phenomenon in our model for ALD. As shown in Figure 2(a), immunohistochemical staining for protein-SSG reveals a marked increase in zone 3 hepatocyte staining. Although the impact of this modification is unknown, current theories propose protein-SSG to be a protective mechanism, shielding critical cysteine residues from more permanent oxidative modifications, that is, 4-HNE [37]. 

Mechanisms behind protein-SSG remain unknown; however, recent reports have proposed a role for the glutathione s-transferase (GST) and glutaredoxin (GRx) family of enzymes [38, 39]. We, therefore, measured these enzymes in our disease model. As shown in Figures 2(b) and 2(c), a significant increase in pan-GST and GRx activities was observed in the ethanol-fed mice following 6 weeks, while no significant difference was observed at weeks 1 or 3. The expression of these enzymes was also assessed via immunoblotting, revealing no significant differences in protein expression (data not shown). These data demonstrate, for the first time, protein-SSG as a consequence of sustained ethanol ingestion and provide novel avenues for research in the field of ALD.

### 3.5. Ethanol Ingestion Leads to Increased Protein Ubiquitination

**Figure 3:**
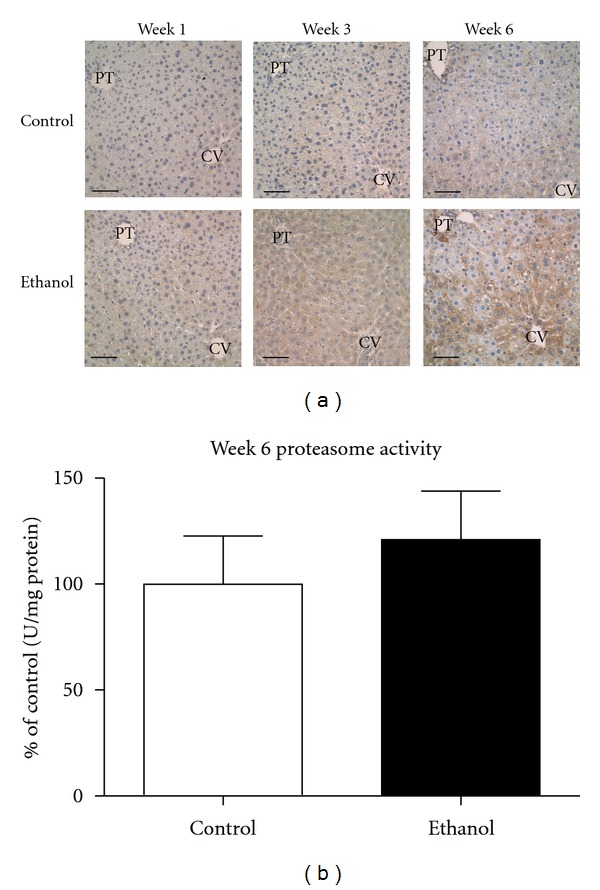
Early-Stage ALD leads to a significant increase in protein ubiquitination. (a) Immunohistochemical analysis reveals marked staining of ubiquitinated proteins in zone 3 and zone 2 of ethanol-fed mice following 6-week ingestion. PT: portal triad; CV: central vein. (*n* = 4 pairs; **P < 0.01). Magnification, 400x; Scale bar represents approximately 50 *μ*m. (b) Hepatic proteasome activity revealed no change at the week 6 time point (*n* = 6 pairs; **P* = n.s.*).

The effects of ethanol consumption and 4-HNE on proteasome activity have been previously documented [40, 41]. Immunohistochemical staining presented in Figure 1(c) reveals a significant increase in 4-HNE protein adducts, suggesting a mechanism for altered protein folding following sustained ethanol consumption. To investigate the impact of sustained oxidative stress on protein folding in this model, immunohistochemical staining was performed using antiubiquitin antibodies to target misfolded proteins targeted for degradation. As shown in Figure 3(a), marked accumulation of ubiquitinated proteins was found throughout zones 2 and 3 in ethanol-consuming mice following 6 weeks. These data suggest an increase in protein misfolding and/or erred protein load as noted by an increasing need for protein disposal. Given the large increase in protein ubiquitination at the week 6 time point, proteasome activity was quantified to determine if the effects on protein ubiquitination were due to altered disposal or increased protein misfolding. As shown in Figure 3(b), no significant alterations in proteasome activity were found between the week 6 control and ethanol-fed mice. These data collectively suggest an increase in protein misfolding following ethanol ingestion, with large quantities of tagged protein being observed during pathologic situations. Given these observations, it was our goal to investigate the role of the UPR in our model for early-stage ALD.

### 3.6. No Evidence for UPR Activation in Early-Stage ALD

**Figure 4:**
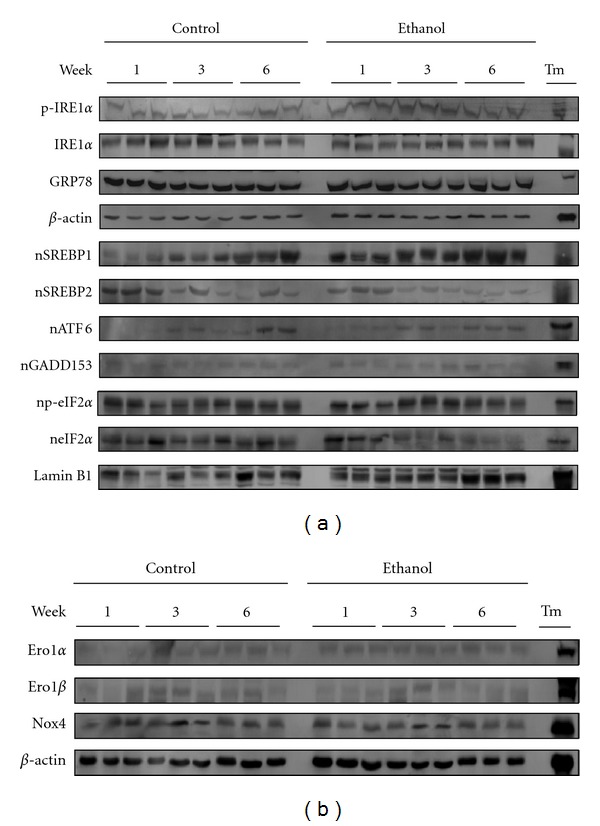
Western blotting reveals a disconnect between the UPR and oxidative stress in the pathogenesis of ALD. (a) Standard western blotting shows a lack of activation in classical UPR signaling cascades at any time point. (b) No induction of the Ero1 proteins or Nox4 was observed, confirming a lack of UPR-induced oxidative stress. *β*-actin was utilized as a loading control for total liver extracts while Lamin B1 was used as a nuclear loading control.

UPR signaling is not only limited to protein expression, as these pathways remain extremely complex and reliant on protein localization and posttranslational modifications [42]. To assess these parameters, immunoblotting was conducted to evaluate UPR protein expression, localization and phosphorylation status. As a positive control, a liver homogenate was utilized from a mouse, which received an intraperitoneal injection of tunicamycin (Tm). As shown in Figure 4(a), the expression of the hallmark indicator for the UPR, glucose regulated protein 78kDa (Grp78), showed no change in expression at any time point. The activation of IRE1*α*, as measured by the relative expression level of its phosphorylated form (p-IRE1*α*), was also observed indicating no major role for this pathway following ethanol ingestion. To investigate the PERK pathway, both total eIF2*α* and activated eIF2*α* (p-eIF2*α*) were assessed. No significant activation of this pathway was observed (Figure 4(a)). Finally, nuclear localization of ATF6 (nATF6) is the major indicator for the activation of this pathway following initiation of UPR signaling. As shown in Figure 4(a), no change in nATF6 was observed between control and ethanol-fed mice, despite a potent activation of this protein following Tm injection. 

The ER stress response has been shown to lead to a host of cellular responses, including steatosis [43, 44]. Relevant to the pathologies associated with early-stage ALD, lipogenic mediators initiated by UPR signaling were investigated (Figure 4(a)). No significant change in the nuclear localization of SREBP1 (nSREBP1) or nSREBP2 was observed between control and ethanol-fed mice at any time point. Although a trending increase in SREBP1 activation was observed, blot densitometry revealed no significant difference among treatment groups (data not shown). 

Previous reports have shown a clear association between the ER stress response and oxidative stress. This link is thought to occur through the induction of the Ero1 proteins (Ero1*α* and Ero1*β*) and Nox4. To investigate a possible separation between these responses, the expression of Ero1*α*, Ero1*β* and Nox4 was assessed via immunoblotting. As shown in Figure 4(b), no significant change in expression was observed, indicating that the observed oxidative stress associated with early-stage ALD precedes the involvement of UPR signaling. 

## 4. Discussion

Previous work has supported roles for oxidative stress and ER stress in the etiology of ALD [4, 5, 8, 13, 14, 45, 46]. These pathological processes are intrinsically linked, with each capable of activating the other [16]. In order to elucidate the mechanisms involved in the initiation of ALD hepatopathy, our aim was to focus on the earliest initiating stages of the disease where therapeutic intervention is typically most effective. This 6-week Lieber-DeCarli model for early-state ALD represents the earliest initiating stages of disease progression as shown through the documented sustained ingestion of substantial quantities of alcohol resulting in significant elevations in plasma ALT, liver triglycerides and increased liver  :  body weight ratio. Histological analyses revealed a marked increase in pan-lobular hepatic lipid accumulation consistent with the early signs of steatosis. Collectively, these data demonstrate a reliable model for early-stage ALD, which allowed for further investigation into the parameters associated with the initiating stages of disease progression.

Oxidative stress is a well-recognized outcome of chronic ethanol ingestion and is likely to play a major role in ethanol-mediated liver damage [4, 5, 8]. Previous works utilizing N-acetylcysteine have demonstrated a protective role for antioxidant therapies in ethanol-mediated liver injury [47, 48]. These studies revealed a significant decrease in ALT measurements, steatosis, and TBARS content. While these data do not definitively prove that oxidative stress is responsible for the early-initiating stages of disease development, a strong correlation can be established. Data presented here reveals a significant decrease in GSH following 6 weeks of ethanol consumption, indicating a decrease in the overall antioxidant capacity of the liver at this time point. Further, a significant decrease in the activity of CBS was observed at the week 6 time point, demonstrating a potential decrease in the GSH precursor cysteine. Previous work conducted on CBS has shown a decrease in enzymatic activity following other hepatic insults such as a methionine-choline-deficient diet and other cellular stresses [49, 50]. We postulate that the effects on CBS activity in the control animals may be due to the high fat content in the control diet. More importantly, the effects of this diet on CBS activity appear to be exacerbated following ethanol consumption. The availability of cysteine is often the rate-limiting determinant in GSH biosynthesis, and these effects on CBS activity offer a potential novel mechanism for the observed decrease in GSH following sustained ethanol ingestion. 

The decrease in GSH was consistent with an increase in lipid peroxidation, as demonstrated by increased staining of 4-HNE modified proteins in the livers of ethanol-fed mice. Previous reports by Backos et al. have highlighted the effects of 4-HNE on the activity of GCL utilizing cultured cells; GCL activity was found to be significantly increased following treatment with 4-HNE, despite a significant depletion of cellular GSH [30]. Our findings revealed a similar result, where GCL activity was found to be significantly increased despite no change in the overall expression of the enzyme. Although the effects of 4-HNE on GCL activity are noted, the precise role of aldehyde adduction in the alcoholic liver remains to be fully characterized. 

Following 6 weeks of ethanol ingestion, hepatic pan-lobular steatosis is observed, with larger lipid droplets present throughout zones 1 and 2. These macrosteatotic vesicles are likely contributing to the increased lipid peroxidation observed, as demonstrated by staining with 4-HNE throughout these zones. It should be noted as well that CYP2E1 is found almost exclusively throughout zone 3 and is completely absent in zones 1 and 2. This may, in part, provide a likely explanation for the observed increase in zone 3 staining of protein-SSG, whereby these modifications act to protect critical thiol residues. Oxidative stress occurring in zone 3 has been documented by numerous other groups; however, the staining of ubiquitinated proteins in this region remains a novel finding. It is conceivable that the increased oxidative stress in zone 3 is resulting in other damaging oxidative modifications stemming from CYP2E1-mediated free radical generation. At the current time, the precise mechanisms and rationale behind the increased ubiquitin staining in zone 3 are unknown. Reports from our laboratory utilizing the Lieber-DeCarli model in rats, however, have identified the ER-resident protein disulfide isomerase (PDI) to be a target for modification by 4-HNE *in vivo*; this leads to a decrease in enzymatic activity and impaired protein folding [51]. Collectively, these data suggest a potential mechanism for oxidative-stress-induced UPR signaling in rodent models for ALD [51, 52].

In the past decade, ER stress has been associated with an increasing number of hepatic disease states, most notably ALD [10, 53]. Previous research in mice by Ji et al. has suggested a role for ER stress in an intragastric model of ALD [13, 14, 45]. However, the intragastric model represents a model for more severe pathologies of ALD, as shown by a roughly 8-fold increase in serum ALT (versus an approximate doubling here) and a significant increase in inflammation (inflammation was not seen in our model). The transition from steatosis to steatohepatitis is considered to be the critical pathogenic determinant in ALD furthering a need for therapeutic intervention during the initiating stages of disease progression [20]. Recent reports have suggested an association between the inflammatory response and UPR signaling, outlining a mechanism similar to that with oxidative stress [54–57]. Consistent with early-stage ALD, our data demonstrate clearly that the inflammatory response does not precede disease pathogenesis and UPR induction in this model.

To further understand the mechanisms behind ALD progression, our research focused on the relative pathogenic contributions of ER stress and oxidative stress during the initiating stages of the disease. Previous work in rodent models of ALD has hypothesized a role for the ER stress response in the activation of lipogenic pathways. This was shown to occur through SREBP1 activation and this mechanism was thought to be a major contributor to the observed hepatic steatosis [13, 45]. To investigate these reports, our studies utilized the Lieber-DeCarli model for ALD. Immunoblotting for both hallmark and lipogenic UPR signaling cascades revealed no significant activation during our time course model for ALD despite the generation of significant pathologies, such as increased lipid accumulation and serum ALT levels. Taken together, these data validate a minimal role for the UPR during the development of ethanol-induced steatosis in a murine model for early-stage ALD.

Oxidative stress has gained considerable attention as a possible mechanism for the induction of various cellular responses, including the ER stress response [8, 15–19, 58]. Additionally, UPR signaling has been shown to lead to induction of the Ero1 proteins and Nox4, furthering the oxidative stress burden in the cell. This oxidative protein folding relay has been estimated by Tu and Weissman to account for up to 25% of total cellular ROS [59]. This creates a vicious cycle of cellular derangements, where each response propagates the other. Regarding the alcoholic liver, UPR signaling and oxidative stress have been intimately associated with the progression of ALD. To date, these responses have not been investigated in relation to the initiation of ALD and therefore the relative role each has during early-stage ALD is not understood.

Our data demonstrate a clear delineation between the ER stress response and oxidative stress in early-stage ALD, indicating that oxidative stress is a primary initiating factor responsible for the progression of ALD. The observed increase in protein glutathionylation demonstrates a potential involvement for this posttranslational modification following increased oxidative stress and provides interesting avenues for research in this field. The effects of oxidative stress in our model were determined to be independent of UPR induction, indicating that the ER stress response may play a pivotal role during more advanced stages of disease progression.

## Figures and Tables

**Table 1:** Composition of ethanol and isocaloric diets. Values are presented as a percentage of the total caloric intake.

Parameter	Week 1		Week 3		Week 6	
	Control	Ethanol	Control	Ethanol	Control	Ethanol
Fat-derived calories (%)	45.0	45.0	45.0	45.0	45.0	45.0
Protein-derived calories (%)	15.0	15.0	15.0	15.0	15.0	15.0
Maltose-dextrin-derived calories (%)	40.0	29.2	40.0	18.5	40.0	8.2
Ethanol-derived calories (%)	N/A	10.8	N/A	21.5	N/A	31.8

**Table 2:** Significant signs of alcohol-mediated liver injury are achieved following 6 weeks of ethanol ingestion. Blood ethanol concentration (BEC) is reported as mg/dL; ethanol ingestion results in a significant increase in liver-body weight ratio as early as 3 weeks; plasma ALT activity is significantly elevated after week 6. Values represent the average ± standard error of the mean (*n* = 12, *P < 0.05, **P < 0.01, ***P < 0.001).

Parameter	Week 1		Week 3		Week 6	
	Control	Ethanol	Control	Ethanol	Control	Ethanol
Food	15.031 ± 0.263	16.510 ± 0.302^∗∗∗^	15.151 ± 0.181	16.389 ± 0.183^∗∗∗^	16.994 ± 0.205	17.778 ± 0.326^∗∗^
Consumption/day						
(mL/Day)						
BEC (mg/dL)	N/A	20.848 ± 0.367	N/A	32.577 ± 6.293	N/A	245.383 ± 33.747
Body weight (g)	25.667 ± 0.607	25.000 ± 0.663	26.900 ± 0.688	26.392 ± 0.601	31.017 ± 0.752	25.783 ± 0.647^∗∗∗^
Liver weight (g)	1.102 ± 0.036	1.113 ± 0.031	1.086 ± 0.035	1.190 ± 0.034	1.148 ± 0.038	1.123 ± 0.036
Liver/body weight	0.043 ± 0.001	0.045 ± 0.000	0.040 ± 0.000	0.045 ± 0.001^∗∗^	0.038 ± 0.001	0.044 ± 0.001^∗∗∗^
Serum ALT activity (U/L)	15.698 ± 1.076	14.744 ± 0.803	11.332 ± 1.175	10.090 ± 0.808	18.411 ± 4.612	42.985 ± 4.523^∗∗^

**Table 3:** Ethanol ingestion leads to decreased GSH and altered GSH metabolism. Reduced GSH is significantly decreased following ethanol consumption; the activities of GSH cycling and metabolizing enzymes show significant fluctuations, consistent with sustained oxidative stress (*n* = 6; *P < 0.05, **P < 0.01, ***P < 0.001).

Parameter	Week 1		Week 3		Week 6	
	Control	Ethanol	Control	Ethanol	Control	Ethanol
GSH (*μ*mol/g tissue)	2.667 ± 0.145	2.421 ± 0.134	2.283 ± 0.065	2.046 ± 0.185	3.367 ± 0.239	2.516 ± 0.157^∗^
GSSG (*μ*mol/g tissue)	0.238 ± 0.012	0.169 ± 0.006	0.100 ± 0.008	0.118 ± 0.012	0.173 ± 0.006	0.155 ± 0.008
GR activity (U/mg protein)	0.981 ± 0.019	0.958 ± 0.027	0.890 ± 0.040	0.850 ± 0.020	0.879 ± 0.028	1.149 ± 0.047^∗∗^
GCL activity (U/mg protein)	0.899 ± 0.042	1.230 ± 0.025^∗∗∗^	1.008 ± 0.032	1.154 ± 0.063	1.029 ± 0.052	1.449 ± 0.083^∗∗∗^
CBS activity (U/mg protein)	10.335 ± 1.285	8.459 ± 0.648	9.136 ± 0.873	9.415 ± 0.554	4.931 ± 0.224	3.549 ± 0.0812^∗∗∗^

## Abbreviations

ALD: Alcoholic liver disease
CYP2E1: Cytochrome P450 2E1
ROS: Reactive oxygen species
Nox: NAD(P)H oxidase
4-HNE: 4-Hydroxy-2-nonenal
PDI: Protein disulfide isomerase
ERAD: ER-associated degradation
UPR: Unfolded protein response
SREBP: Sterol regulatory element-binding protein
GADD153/CHOP: Growth arrest and DNA damage 153
Grp78/94: Glucose regulated protein 78/94
Ero1: ER oxidoreductase 1
ALT: Alanine aminotransferase
TBARS: Thiobarbituric acid reactive substances
MDA: Malondialdehyde
GR: Glutathione reductase
GST: Glutathione S-transferase
GPx: Glutathione peroxidase
GSH: Glutathione
CBS: Cystathionine *β*-synthase
GCL: Glutamate-cysteine ligase
H&E: Hematoxylin & eosin
GR: Glutathione reductase
Protein-SSG: Protein glutathionylation.
